# Chemical Extraction and Gastrointestinal Digestion of Honey: Influence on Its Antioxidant, Antimicrobial and Anti-Inflammatory Activities

**DOI:** 10.3390/foods10061412

**Published:** 2021-06-18

**Authors:** Marta Alevia, Sandra Rasines, Leire Cantero, M. Teresa Sancho, Miguel A. Fernández-Muiño, Sandra M. Osés

**Affiliations:** Department of Biotechnology and Food Science, University of Burgos, Pza. Misael Bañuelos s/n, 09001 Burgos, Spain; maa1011@alu.ubu.es (M.A.); srm0041@alu.ubu.es (S.R.); lcantero@ubu.es (L.C.); smoses@ubu.es (S.M.O.)

**Keywords:** honey, in vitro digestion, phenolic extract, antioxidant activity, antimicrobial activity, anti-inflammatory activity

## Abstract

The effect of chemical extraction and in vitro digestion of different kinds of honey on bioactive compounds (total phenolic compounds and flavonoids) and biological activities (antioxidant, antimicrobial and anti-inflammatory) was investigated. The antioxidant activity was evaluated against three radicals (ABTS^•+^, ROO^•^, ^•^OH), and the antimicrobial activity was studied against five bacteria (*Staphylococcus aureus*, *Listeria monocytogenes*, *Escherichia coli*, *Streptococcus mutans* and *Pseudomona aeruginosa*) and one yeast (*Candida albicans*). The results show that in comparison with raw honeys, the methanolic extracts exhibited lower values for phenols, flavonoids and antioxidant activity and higher anti-inflammatory and antimicrobial activities against *L. monocytogenes.* The higher anti-inflammatory activity indicates a possible use of dried honey extracts in the pharmaceutical and cosmetic industries. The digested honeys showed higher total phenolics and higher antioxidant activity than the pre-digested honeys, as well as higher antimicrobial activity against *S. aureus* and *L. monocytogenes*, which underlines the possible antioxidant and antimicrobial effects of honey in the human body after the digestion process.

## 1. Introduction

Honey is a complex natural product produced by bees from flower nectar or tree and plant exudates, usually involving plant-sucking insects. This product has been used since ancient times mainly as a sweetening agent but has also been used for its therapeutic capacity [1]. Honey is a mixture of carbohydrates, especially glucose and fructose (60–85%), water (14–22%) and minority compounds including phenolic compounds, minerals, proteins, enzymes, free amino acids, organic acids and vitamins [2]. Phenolic compounds, especially phenolic acids and flavonoids, are important for their functional properties, and their concentration shows a large variation depending on the botanical origin of honey [3].

Due to its composition, honey was traditionally used for medicinal applications. Its beneficial properties include mainly antibacterial, anti-inflammatory and antioxidant activities [4,5]. Honey’s total phenolic compounds and the biological properties of honey have been extensively studied in different types of honey from all over the world [6,7,8,9]; however, there are few studies that assess the biological activities of their alcoholic extracts where these phenolic compounds are concentrated, and which can be used as additives in food, pharmaceutical and cosmetic industries.

There are also few studies investigating the effect of digestion on honeys’ bioactive compounds. This information is very important, as bioactive compounds that are not degraded during digestion (bioaccessibility) may be available for absorption, especially in the gut, promoting biological action (bioavailability) [10]. In vitro bioaccessibility studies are useful to study the release of bioactive compounds from honey and their stability under gastrointestinal conditions [3]. An extensive literature review only showed six papers that studied the effect of in vitro digestion on phenolic compounds, antioxidant and antimicrobial activities of honey [3,11,12,13,14,15] and one review [16]. All the mentioned papers roughly followed the same protocol. In none of them was the simulation of the oral phase carried out, mainly because honey is not a solid food, and during its production by bees, this process is partially performed, although it may be interesting to study its effect on honey. The main components of honey are monosaccharides (fructose and glucose) that are absorbed with no modification. Digestion in the large intestine may be of interest for some minority components with functional activities. The major difference between the honey-digestion studies was related to the honey dilution before digestion. Cianciosi et al. [11] used 10 g of honey dissolved in 100 mL miliQ water, Daglia et al. [12] and Mannina et al. [15] used 20 g in 10 mL water, and O’Sullivan et al. [13] used 0.5 g in 10 mL water. It is likely that those different concentrations could influence the values of the analyzed activities, making it difficult to compare results.

The aim of this study was to analyze, in quantitative–qualitative fashion, total phenols and flavonoids, and evaluate the antioxidant, anti-inflammatory and antimicrobial activities of 15 honeys before and after in vitro digestion, as well as their methanolic extracts in order to determine whether gastrointestinal digestion and/or chemical extraction can modify the functional properties of raw honeys.

## 2. Materials and Methods

### 2.1. Materials

Ethanol, methanol, sodium carbonate, sodium chloride, sodium hydroxide, potassium persulfate, Fe(NH4)_2_(SO_4_)_2_, EDTA, acetic acid, formic acid, hydrochloric acid, p-dimethylaminobenzaldehyde (DMAB), Baird Parker agar base (BP) and Trypone bile x-glucoronide agar (TBX) were acquired from the VWR International Eurolab, part of Avantor (Llinars del Vallés, Cataluña, Spain). Gallic acid (GA), NaNO_2_ and catechin (Cat) were acquired from Panreac (Barcelona, Spain). AlCl_3_ and fluorescein sodium were acquired from Fluka Chemie GmbH, part of Sigma-Aldrich (Buch, San Galo, Switzerland). Na_2_HPO_4_, NaH_2_PO_4_ and Microinstant Listeria Agar base (Ottaviani and Agosti) (MLA) were acquired from Scharlab (Sentmenat, Spain). Potassium tetraborate, sodium benzoate and uric acid (UA) were purchased from Alfa Aesar, part of Thermo Fisher (Kanderl, Germany). α-amilase, pepsin, pancreatin, bile salts, Folin–Ciocalteu reagent, 2,2′-azino-bis (3-ethyl-benzothiazoline-6-sulphonic acid) (ABTS), 6-hydroxy-2,5,7,8-tetramethylchroman-2-carboxylic acid (Trolox, T), H_2_O_2_, thiobarbituric acid, KH_2_PO_4_, 2,2′-azobis (2-amidopropane) dihydrochloride (ABAP), *N*-acetyl-d-glucosamine (NAG), hyaluronic acid sodium salt (HA) from *Streptococus equi*, bovine serum albumin (BSA), hyaluronidase from bovine testes type IV-S (1400 U/mL) and glycerol were acquired from Sigma-Aldrich part of Merck (Steinheim, Germany). Nutrient broth No. 2 (NB), malt extract broth (MEB), brain–heart infusion (BHI), agar technical NO. 1 and Ringer solution were acquired from Oxoid, part of Thermo Fisher (Basingstoke, Hampshire, UK). Water was deionized using a Milli-Q water purification system (Millipore, Bedford, MA, USA). Spectrophotometric analysis was performed on a Varian Cary Bio 400 spectrophotometer (Varian, part of Agilent Technologies, Santa Clara, CA, USA) and the fluorimetric analysis by using a fluorometer Varioskan LUX microplate reader (Thermo Fisher, Kanderl, Germany).

### 2.2. Samples

The study was conducted on 15 honeys of different botanical and geographical origins collected in autumn 2016 in Castilla y León (Spain) from individual apiaries. Melissopalinology [17,18,19] and sensory analysis [20,21] showed that there were 4 honeydew honeys (RH1-RH4), 3 viper’s tail honeys (*Echium* spp.) (RH5-RH7), 5 heather honeys (*Calluna vulgaris* and *Erica* spp.) (RH8-RH12) and 3 broom honeys (Leguminosae *Genista* spp.) (RH13-RH15) (Table 1).

### 2.3. Chemical Extraction

Methanolic extracts of honey (ME1-ME15) were obtained following the method proposed by Baltrušaityte et al. [22]. 25 g of honey was dissolved in 250 mL of distilled water, adjusting the pH to 2 with concentrated HCl. The diluted sample was passed through a column (300 × 15 mm) with Amberlite XAD-2 resin (Supelco, Bellefonte, PA, USA), preconditioned with methanol and distilled water. The column was then washed with 250 mL of acidified water (pH 2 with HCl) and 300 mL of neutral distilled water to remove sugars and other interferents. Finally, the phenolic compounds were eluted with 250 mL methanol and the phenolic extracts were evaporated to dryness using a rotary evaporator. They were then re-dissolved in 25 mL of HPLC-grade methanol. In order to perform all the analyses foreseen for this study for each honey, 2 extractions were performed, and both extracts were then homogenized. The methanolic extracts were stored at −20°C until analysis.

### 2.4. Simulated In Vitro Digestion

Samples were first subjected to oral digestion in accordance with the procedure performed by Pastoriza et al. [23]. Honey (1.25 g) was weighed and dissolved in 10 mL of milli-Q water. Then, 250 µL of α-amylase solution (3.25 mg of α-amylase dissolved in 2.5 mL of CaCl_2_ 1mM pH 7) was added. The mixture was incubated in a water bath at 37 °C for 30 min, shaking at 110 oscillations/min. Following this step, gastric digestion and intestinal digestion were performed using the procedure adapted by Rufián-Henares and Delgado-Andrade [24]. For gastric digestion, the pH was adjusted to 2 with 6N HCl and 1 mL of pepsin solution (0.4 g of pepsin dissolved in 2.5 mL of 0.1M HCl) was added. The samples were incubated in a water bath at 37 °C for 2 h with shaking at 110 oscillations/min. For the intestinal phase, the pH was raised to 6 with 1M NaHCO_3_ and a solution of pancreatin and bile salts (0.1 g pancreatin + 0.625 g bile salts dissolved in 25 mL 0.1M NaHCO_3_) was added. The pH was subsequently adjusted to 7.5 with 1M NaHCO_3_ and the samples were incubated in a water bath at 37 °C for 2 h with shaking at 110 oscillations/min. After gastrointestinal digestion, digestive enzymes were inactivated by heat treatment for 4 min at 100 °C. Samples were cooled and centrifuged at 3550 rpm for 1 hour at 4 °C to separate bio-accessible (soluble) and non-accessible (insoluble) fractions. Subsequent analyses were performed only on the soluble fraction (DH1-DH15), due to the minimal amount of insoluble fraction obtained. Blank digestion was performed with miliQ water, subtracting its value from those obtained in the samples to eliminate the interaction of the enzymes used in the digestion.

### 2.5. Total Phenolics and Flavonoids Content

Total phenolics were measured in the raw honey samples (RH1-RH15), in the methanolic extracts (ME1-ME15) and after in vitro digestion (DH1-DH15). For the analysis of the raw honey samples, a dilution to 100 mg/mL with distilled water was performed in advance. Then, all samples were filtered with Whatman No. 40 paper. For the analysis of methanolic extracts and digested honey samples, the extract or the soluble fraction obtained after in vitro digestion was directly taken.

Total phenolics content was determined using the Folin–Ciocalteau reagent [25]. A unit of 0.5 mL of sample was mixed with 2.5 mL of 0.2 N Folin–Ciocalteau reagent. After 5 min, 2 mL of Na_2_CO_3_ (75 g/L) was added. After incubation of the mixture for 120 min in the dark at room temperature, the absorbance was measured at 760 nm. The standard for the calibration curve was GA (1–300 mg/L) prepared with 70% ethanol. Results were expressed as mg GA/100 g honey.

The total flavonol content was assessed by measuring the colour of aluminium-flavonoid complexes in alkaline medium [26]. 0.15 mL NaNO_2_ (5% *w*/*v*) was added to each sample (0.5 mL). After 5 min, 0.25 mL of AlCl_3_ (2% *w/v*, in methanol) was added. Within 6 min, the mixtures were neutralized with 0.25 mL of 1M NaOH. The absorbance was read at 510 nm against a water blank for RH and DH and a methanol blank for ME after 10 min at room temperature. Cat (1–80 µg/mL) was the standard used for the calibration curve. Results were expressed as mg Cat/100 g of sample.

### 2.6. Antioxidant Activities

Three different assays with different free radicals were used to assess the antioxidant activities of RH, ME and DH: Trolox equivalent antioxidant capacity (TEAC), antioxidant activities against hydroxyl (AOA) and oxygen radical absorbance capacity (ORAC).

TEAC by ABTS procedure: TEAC was assayed according to the process described by Sancho et al. [27] in honeys, extracts and fractions obtained after in vitro digestion. ABTS^•+^ was generated by oxidation of a 7 mM ABTS stock solution with 2.45 mM potassium persulphate (1:1) in the dark for 16 h. Then, the ABTS^•+^ solution was diluted to obtain ana absorbance between 0.70 and 0.80 at 734 nm. ABTS^•+^ solution (1490 µL) was added to 10 µL of sample (RH (500 mg/mL), ME or DH), standard or blank (distilled water for honeys and methanol for honey extracts and digests). The standard calibration curve was plotted using TE (0.625–3 mM) as standard, calculating the percentage inhibition after 6 min as follows: % inhibition = [(Ab − As)/Ab × 100], where Ab = absorbance of the blank, As = absorbance of the sample or standard. The results were given in µmol Trolox Equivalents (TE)/100 g honey.

Radical-scavenging activity on hydroxyl radicals (AOA assay): AOA was evaluated by the Koracevic et al. procedure [28]. Samples (10 µL) (RH 750 mg/mL, ME and HD) were mixed with 490 µL of sodium phosphate buffer (0.1 M, pH 7.4), 500 µL of sodium benzoate (0.01 M), 200 µL of FeSO_4_-EDTA (2 mM) and 200 µL of hydrogen peroxide (0.01 M). Samples were then incubated for 1 h at 37 °C, after which the reaction was stopped by addition of 1 mL acetic acid (20%). Subsequently, 1 mL of thiobarbituric acid (0.8% *w/v*) in NaOH (50 mM) was added. The solution was boiled for 10 min and then cooled on ice. The absorbance of all samples was measured at 532 nm against distilled water. Each sample (A_1_) had its own control (A_0_), in which acetic acid (20%) was added before Fe-EDTA and H_2_O_2_. Negative controls (K_1_ and K_0_) were prepared by substituting the samples with phosphate buffer. Uric acid 1 mM in NaOH (5 mM) was used as standard (U_1_ and U_0_). Antioxidant activity was determined as mmol uric acid (UA)/100 g honey = f × (CU) × (K − A)/(K − U), where f is the dilution factor, CU is the concentration of uric acid (1 mM), K is the absorbance of the control (K_1_ − K_0_), A is the absorbance of the sample (A_1_ − A_0_) and U is the absorbance of the uric acid solution (U_1_ − U_0_).

Oxygen radical absorbance capacity (ORAC): ORAC values were assessed by a modified assay based on the Huang et al. procedure [29], using a 96-well white plate (Greiner Bio-one, San Sebastián de los Reyes, Madrid, Spain). Three microlitres of fluorescein (4.1 µM) was added to 187 µL of sample (RH 100 mg/mL, ME and DH), buffer solutions (75 mM sodium phosphate, pH 7.4) or TE (0.2 µM) in a 96-well white plate at 37 °C for 5 min. ABAP (10 µL, 0.37 M) was added to the mixtures and the fluorescence (excitation wavelength 485 nm and emission wavelength 520 nm) was then recorded every 5 min for 90 min. Results were expressed as µmol TE per gram of honey = ((Sample area − Target area)/((Trolox equivalent area − Target area)/µmol trolox)).

### 2.7. Anti-Inflammatory Activity

Anti-inflammatory activity was evaluated using a modified hyaluronidase inhibition assay [30]. Hyaluronidase activity was defined as one unit (U) of hyaluronidase catalyzing the release of 1 µmol of *N*-acetyl-d-glucosamine (NAG) per minute under specified conditions. A hyaluronic acid sodium salt stock solution (5 mg/mL) was prepared and stored at 4 °C. For the assay, 70 µL of hyaluronic acid stock solution and 100 µL of buffer (0.2 M sodium formate, 0.1 M NaCl and 0.2 mg/mL BSA, pH 3.68) were added to 200 µL of milliQ water and 50 µL of sample (RH 750 mg/mL, ME and DH). The mixture was heated at 37 °C for 10 min. The reaction was then initiated by adding 50 µL of hyaluronidase type IV-S (1400 U/mL) prepared in 0.9% NaCl, incubating the mixture in a water bath for 1 h at 37 °C and stopping the enzymatic reaction by addition of 100 µL of 0.8 M potassium tetraborate. The samples were then incubated for 3 min in a boiling water bath and then cooled to room temperature. After adding 750 µL of DMAB, the tubes were incubated for 20 min at 37 °C and then the absorbance at 586 nm was read against a blank sample, in which both enzyme and sample were replaced by buffer. Calibration curves were drawn with standard solutions of NAG (in the range between 0 and 2 µmol per test). Percent inhibition of hyaluronidase was calculated as follows: % inhibition = (A − B)/A × 100, where A was μmol of NAG in the positive control and B was µmol of NAG from each sample reaction.

### 2.8. Antimicrobial Activity

*Escherichia coli* CECT 99, *Listeria monocytogenes* CECT 934, *Pseudomonas aeruginosa* CECT 108, *Streptococcus mutans* CECT 479, *Staphylococcus aureus* CECT 435, as well as a yeast, *Candida albicans* CECT 1394, were used in the assays. The cells were grown in NB for *S. aureus*, *Ps. aeruginosa* and *E. coli* and in BHI for *L. monocytogenes* and *St. mutans* for 24 h at 37 °C and in SEM for *C. albicans* for 24 h at 30 °C. Cell suspensions were diluted in sterile Ringer’s solution to obtain initial cell counts of 6 log cfu/mL.

Minimum inhibitory concentration: antimicrobial activity was evaluated by broth dilution assay. The minimum inhibitory concentration (MIC) and minimum bactericidal concentration (MBC) were estimated using different concentrations for each sample.

For raw honey, 37.5, 18.75, 9.38, 4.69, 2.34, 1.17 and 0.59% (*w/v*) were employed, except for *C. albicans*, where the used concentrations were 45, 41, 37.5, 18.75, 9.38, 4.69 and 2.34% (*w/v*). For ME, 90, 75, 60, 45, 30, 15, 5 and 1% (honey concentration from 450 to 5 mg/100 mL) were used, whereas for DH, 90, 75, 60, 50, 40, 25, 12.5 and 5% (honey concentration between 7.04 and 0.40 mg/100 mL) were used. In the case of DH, it was not possible to use higher honey concentrations, because in the soluble fraction obtained after digestion, the honey concentration was already 7.82% (1.25 g in 16 mL soluble fraction). All dilutions were made with the broth appropriate to each microorganism.

To evaluate antimicrobial activities, sterile 96-well round-bottom polystyrene microtitre plates (Brand, Wertheim, Germany) were employed. For each sample and for each microorganism, 900 µL of each dilution were made up in sterile Eppendorf tubes with broth. As a contamination control, one column was used for each sample. A unit of 180 µL of each Eppendorf dilution was added together with 20 µL of ringer. Additionally, 80 µL of 6 log CFU/mL of each microorganism was mixed with 720 µL of each sample dilution. In each well, 200 µL of the blend was added (three replicates per dilution, eight dilutions). Control wells containing only bacteria and broth (positive control) and bacteria with bleach (negative control) were also prepared. Plates were incubated at 37 °C for 24 h for bacteria and at 25 °C for *C. albicans*. Visible growth (MIC) was determined. The MIC was determined as the minimum concentration at which no growth of the microorganism was visually observed. Then, 10 µL of each well in which bacterial growth was inhibited was plated on the corresponding agar: TBX for *E. coli*, MLA for *L. monocytogenes*, BP for *S. aureus*, nutrient agar for *St. mutans* and *Ps. aeruginosa* and MEA for *C. albicans*, and incubated overnight at 37 °C or 25 °C to record the CMB. The minimum bactericidal concentration was determined as the minimum concentration of honey at which microorganisms did not grow on the agar plates after 24 h at 37 °C/25 °C.

### 2.9. Statistical Analysis

All analyses were performed in triplicate, expressing the results as mean values and standard deviations. A normality test was performed for all parameters. Parametric results were tested using multiple range tests with the LSD test (*p* < 0.05), while non-parametric values were assessed using the Kruskal–Wallis test and box-and-whisker plots. Principal component analysis (PCA) and Pearson correlations were applied to the results. Statgraphics Centurion XVIII statistical software (Statgraphics Technologies, Inc., The Plains, VA, USA) was used.

## 3. Results and Discussion

### 3.1. Total Phenolics and Total Flavonoids

**Figure 1:**
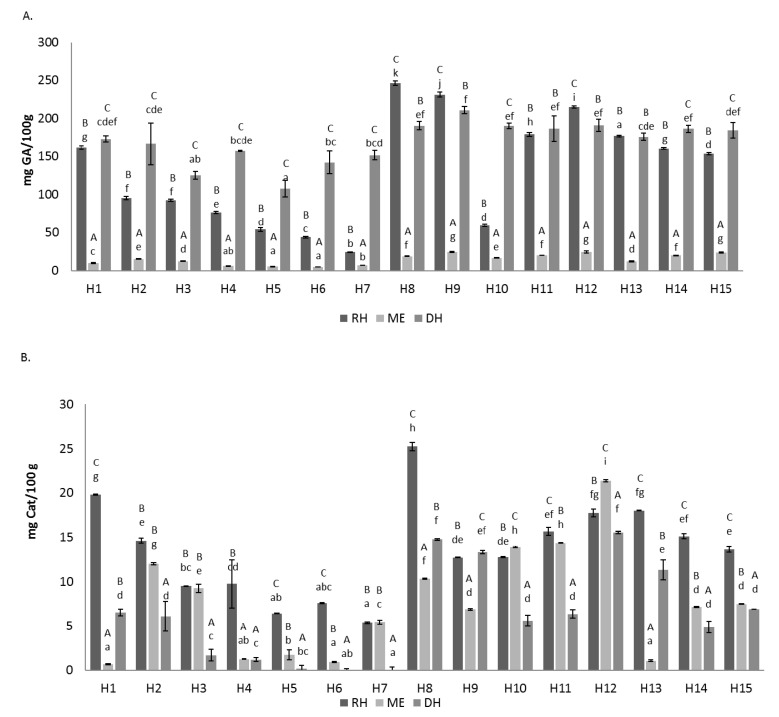
Total phenols (**A**) expressed as mg of gallic acid/100 g of honey and total flavonoids, and (**B**) expressed as mg of catechin/100 g of honey of raw honeys (RH), methanolic extracts (ME) and digestive extract (DH). Error bars represent the standard deviation for each data point. Different capitals letters (A–C) indicate significant differences (*p* < 0.05) between extractions comparing the same sample. Different lowercase letters (a–g) indicate significant differences (*p* < 0.05) between samples with the same extraction.

Total phenolics in raw honey samples ranged between 24 and 242 mg GA/100 g. In methanolic extracts, the variation was between 4.9 and 25 mg GA/100 g. After in vitro digestion, the values ranged between 108 and 211 mg GA/100 g (Figure 1A). Both in raw honeys, extracts and digests, the highest content of total phenolics was obtained in heather honeys, followed by broom honeys and the lowest content was found in viper’s bugloss honeys. The values of the raw honeys were higher than the results provided by literature, where values between 12 and 140 mg GA/100 g were obtained in honeys from different botanical and geographical origins [31,32,33]. Similar to our values were those described by Jara-Palacios et al. [34] in Spanish oak honeydew honeys (50.04–243.86 mg GA/100 g). The results obtained in this work for methanolic extracts were lower than those obtained by Almeida da Silva et al. [35] in samples from the Amazon of Northern Brazil, with a total phenolics content ranging from 17 to 66 mg GA/g extract. The lower values of total phenolics found in MEs in comparison with raw honeys were expected, because the Folin–Ciocalteu method also quantifies other reducing compounds apart from phenolics [36]. In all honeys, the phenolic compounds significantly increased after in vitro digestion, except in the three honeys with the highest phenolic content (M8, M9 and M12), in which after the digestion process, the total phenolics decreased. The blank digestion value was considered in the calculations to remove the interactions that the Folin–Ciocalteu reagent could have with the enzymes used in the digestion. However, the likely interaction of sugars and the Folin–Ciocalteu reagent were not eliminated, which could be the reason for the increase of this result. While Cianciosi et al. [11] showed a six-fold decrease in the amount of total phenolics from raw honey to honey after in vitro digestion and Rodriguez Romero [37] showed a decrease in different phenolic compounds after digestion, Seraglio et al. [3] observed no differences after gastric digestion, but a decrease in total phenolics after duodenal digestion. In the study completed by O’Sullivan et al. [13], there was no significant change in phenolic compounds after in vitro digestion, and in some honeys, they slightly increased, as it occurred in our study. The effect of an in vitro digestion on the phenolic content of a food varies depending on several factors, such as the food matrix and digestion conditions. Therefore, differences between studies are likely to be found [3]. The different results obtained in this study in comparison with those of other published papers would be likely due to the fact that some phenolic compounds could be more sensitive than others to pH changes [11] and to the different phenolic composition of several honeys. Most research was conducted on Manuka honeys, with there being only one study regarding honeydew honeys. Another possible reason for the different results could be the wide variety of concentrations used during digestion in the different works or because in our study, the simulation of the oral phase was carried out, in contrast to other studies.

Regarding total flavonoids, the honeys with the highest values both raw and regarding methanolic extracts and in vitro digestion were heather honeys. Conversely, viper’s bugloss honeys exhibited the lowest values for total flavonoids. The ranges of variation in raw honeys were between 5.4 and 25 mg Cat/100 g, in methanolic extracts between 0.93 and 21 mg Cat/100 g and in in vitro digestions between 0 and 16 mg Cat/100 g (Figure 1B).

A significantly higher flavonoids content was obtained in the raw honey samples than in the methanolic extracts in most honeys, corroborating that honeys’ flavonoids must be measured on alcoholic extracts to remove interferences [27]. Only ME10 and ME12 showed higher values than their correspondent RH, probably because the result depends on the specific flavonoid composition of each sample. The total flavonoids’ content was higher than the data described by Osés et al. [9] in extracts of strawberry tree honeys (2.2–6.8 mg Cat/100 g). After the digestion process, the values of total flavonoids were lower than those of raw honeys in all honeys except in DH 9, where flavonoids slightly increased from 12.71 to 13.33 mg Cat/100 g of honey. Cianciosi et al. [11] showed a very drastic decrease (eight-fold) of flavonoids after digestion, obtaining 6.49 mg Cat/kg honey.

Although there is an increase in the amount of total phenolic compounds after in vitro digestion, there is a decrease in flavonoids, which might be related to the fact that the Folin–Ciocalteu method is not specific for phenolic compounds, but measures different reducing substances (sugars, proteins), whereas the procedure described for flavonoids is more specific for flavonoids such as rutin, luteolin and catechins, although it can also measure some phenolic acids [26].

### 3.2. Antioxidant Activities

**Figure 2:**
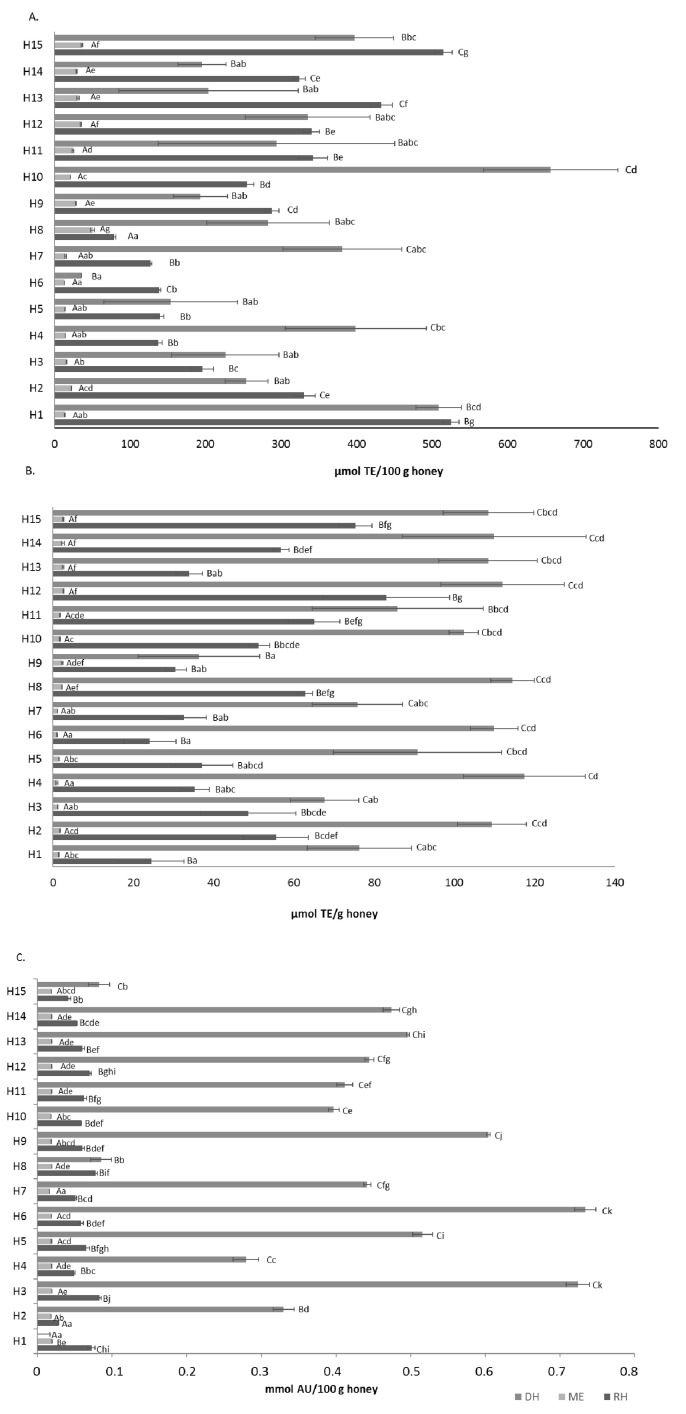
Antioxidant activity of raw honeys (RH), methanolic extracts (ME) and digestive extract (DH) against different radicals (ABTS^•+^ (**A**), ROO^•^ (**B**) and ^•^OH (**C**)). Error bars represent the standard deviation for each data point. Different capitals letters (A–C) indicate significant differences (*p* < 0.05) between extractions comparing the same sample. Different lowercase letters (a–g) indicate significant differences (*p* < 0.05) between samples with the same extraction.

The antioxidant properties of the raw honey samples, extracts and digests were evaluated against different radicals (Figure 2).

The activity against the ABTS^•+^ radical ranged between 79.08 and 525.01 µmol TE/100 g honey. These values were higher than those reported for Slovakian honeys, with values ranging from 180 to 1110 µmol TE/kg and for Cuban honeys, whose results ranged from 210 to 2940 µmol TE/kg [38].

As with the results of total phenolic contents, antioxidant activities were higher in raw honeys and digests than in methanolic extracts. Conversely to the results of other researchers [3,11,13] who reported lower DPPH, FRAP or TEAC activities after digestion, our results showed that the measured antioxidant activities increased in honey digests. However, in other food matrices, an increase in the antioxidant activity of the sample after digestion was claimed to be due to the release of antioxidant compounds from the enzymatic digestion [39]. As for phenolic, different results in our study could be due to the inclusion of the simulation of the oral phase, which was not performed in other studies. It is worth emphasizing the high deviation of the antioxidant activities obtained in honeys after in vitro digestion so that in some cases, no significant differences (*p* > 0.05) were found regarding the results in raw and in vitro digested honeys. The greatest increase in antioxidant capacity after digestion occurred against the hydroxyl radical (AOA), and the smallest increase occurred against the ABTS radical, against which only five of the samples showed greater activity after in vitro digestion. We did not find significant differences among the different honey origins, except to indicate that in general, the results were lower in the viper’s bugloss honeys (both in crude, extract and digestion), and the results were higher in the honeydew and heather honeys.

A significant correlation was observed between total ABTS, ORAC and AOA and total phenolic content (*r* = 0.7287; *r* = 0.7754; *r* =0.4852; *p* < 0.05) assessing all the honey samples and the three different extractions (*n* = 135).

### 3.3. Anti-Inflammatory Activity

**Figure 3:**
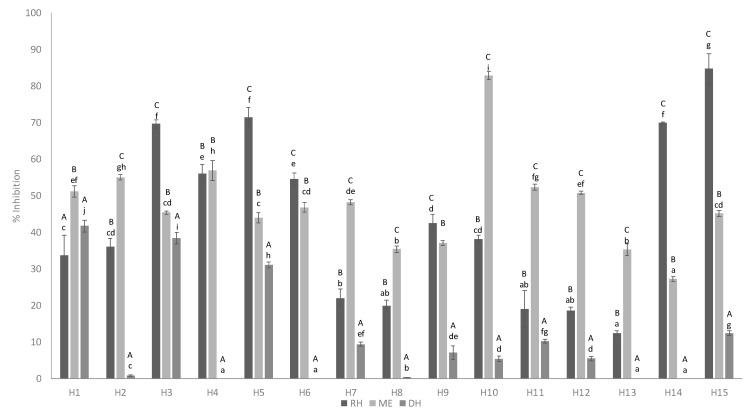
Anti-inflammatory activity of raw honeys (RH), methanolic extracts (ME) and digestive extract (DH). Error bars represent the standard deviation for each data point. Different capitals letters (A–C) indicate significant differences (*p* < 0.05) between extractions comparing the same sample. Different lowercase letters (a–g) indicate significant differences (*p* < 0.05) between samples with the same extraction.

In the raw honeys studied, the hyaluronidase inhibitory activity varied between 12 and 85% for a honey concentration of 750 mg/mL. In the extracts, inhibition rates varied between 27 and 83%. After digestion, inhibition of hyaluronidase fluctuated between 0 and 42% (Figure 3). Literature also showed that the values of anti-inflammatory activity were highly variable depending on the concentration of honey and phenolic compounds [9]. In this case, we observed a similar inhibition trend between the raw honey and the extracts, with a considerably reduced anti-inflammatory activity in the soluble fraction obtained after in vitro digestion, which might be due to the decrease in the compounds responsible for this activity during the honey-digestion process.

### 3.4. Antimicrobial Activity

Results of the antimicrobial activity showed that all honeys exhibited antimicrobial activity against the six microorganisms evaluated. In Table 2, antimicrobial activity data were expressed as % honey, extract or digestive fraction, with the corresponding honey concentration (g/100 mL) in brackets for a better comparison of results.

*S. aureus* was the most sensitive microorganism to the 15 honeys analyzed, obtaining MICs between 4.69 and 9.38%. The yeast *C. albicans* was the most resistant, against which only honey 14 showed a bactericidal (MBC) effect at a concentration of 45%, although all the honeys showed inhibitory activity (MIC = 37.5%). Honey 2 (honeydew) exhibited the lowest inhibitory effect against all microorganisms. The MIC and MBC data of the different honeys coincided except for *L. monocytogenes* and *C. albicans,* whose MBC were higher concentrations than MIC.

The MIC and MBC data obtained against *S. aureus* were comparable to the values obtained by Osés et al. [40]. This study also concluded that honeys with similar botanical origins showed different antimicrobial activities, i.e., no relationship between botanical origin and the inhibitory effect of the honeys against each of the microorganisms could be observed.

Regarding the antimicrobial activity of the methanolic extracts, *L. monocytogenes* was the most sensitive microorganism, while *St. mutans* together with *C. albicans* were the most resistant. The MIC and MBC obtained were extract concentrations between <1 to 15%, corresponding to <5 to 75 g/100 mL. Therefore, for all the microorganisms evaluated, except for *L. monocytogenes,* the MIC was higher in the ME than in RH, and therefore a lower antimicrobial activity was obtained for the extracts in comparison with raw honeys. With regard to *L. monocytogenes*, methanolic extracts showed a greater antimicrobial effect than raw honeys, which could be due to the fact that this microorganism was more sensitive to the phenolic compounds extracted, while the rest of the microorganisms were more sensitive to non-phenolic compounds that are present in raw honey. Previous studies demonstrated the antimicrobial activity of phenolic acids (coumaric, ferulic and syringic) against *L. monocytogenes* [41]. Malheiro et al. [42] also demonstrated that cinnamic acid and cinnamaldehyde and their derivatives significantly inhibit the growth of bacteria such as *E. coli* and *S. aureus*.

Regarding the soluble fraction obtained after in vitro digestion, we observed that it only exhibited antimicrobial activity against *S. aureus* and *L. monocytogenes*, showing MICs between <5 to 40% (0.39–3.13 mg/100 mL) for *S. aureus* and between 40 and >90% (3.13–>7 mg/100 mL) for *L. monocytogenes*. Higher concentrations of these extracts could not be evaluated because in the soluble fraction obtained after digestion, the honey concentration was already 7.82%. The MICs obtained against these two microorganisms were lower than the MICs obtained for the corresponding crude honeys, which could be partly due to the enzymes added during the in vitro digestion, because the digestion blank also showed some inhibition for both microorganisms (60% digestion fraction). However, the samples exhibited a lower MIC (and therefore higher antimicrobial activity) than the blank, so that the antimicrobial activity against *S. aureus* and *L. monocytogenes* exhibited by the soluble fraction obtained after in vitro digestion of honeys is likewise a fact. Mannina et al. [15] reported no antimicrobial activity against *S. aureus* after Manuka honey gastric digestion and a low reduction in the antistaphylococcal activity after gastroduodenal digestion when compared with the undigested sample. Antimicrobial activity of the fraction obtained after digestion was also studied in other foodstuffs obtaining different results. In coffee pulp aqueous extract, antimicrobial activity against *E. coli* and *S. aureus* after in vitro digestion decreased, suggesting that bioactive compounds might degrade during in vitro digestion, there being also a structural disruption conducted by both the digestive enzymes and the low pH in the digestive system. Khochapong et al. [43] and López-Nicolas et al. [44] also obtained a decrease of approximately 10% of antibacterial activity against *E. coli* and *S. aureus* with fruit juices after in vitro digestion. However, Piscopo et al. [45] obtained an increase of the antimicrobial activity of *Feijoa sellowiana* with Bert. fruit extract, after in vitro gastrointestinal digestion.

### 3.5. Principal Components Analysis (PCA)

**Figure 4:**
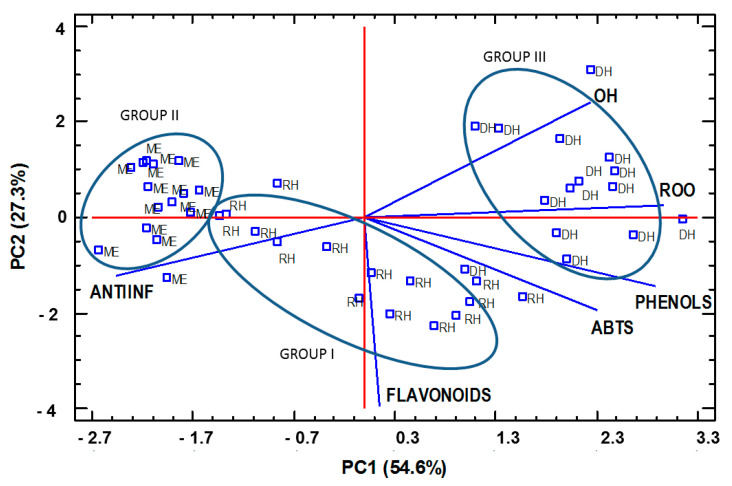
Principal components analysis derivate from phenols compositions, antioxidant and anti-inflammatory activity of RH, ME and DH.

In order to study the influence of both chemical and digestion procedures on the results, phenols, flavonoids, antioxidant and anti-inflammatory activities were analyzed by PCA. The first two components described the 78.9% variance (Figure 4). PC1 included the information about total phenols and antioxidant activity against ROO^•^, while PC2 was defined by flavonoids. Samples with similar scores were positioned closely together. PCA divided the samples into three groups. RH were in the middle (group I), with a higher flavonoids content than the DH and ME. ME were in the left side of Figure 4 (group II), showing a higher anti-inflammatory activity and lower phenols’ contents and antioxidant activities. DH were in the right side (group III) showing higher total phenols contents and higher antioxidant capacities. The fact that ME and DH were on opposite positions is logical, because total phenols actually comprise all reducing compounds, some of which are not extracted with methanol. Conversely, some hydrolysis procedures including in the digestion increase reducing substances that are quantified as total phenols. As expected, there were correlations among the reducing compounds evaluated by Folin–Ciocalteau and the antioxidant activities. However, the anti-inflammatory activity was not related to any of these compounds.

## 4. Conclusions

Heather honeys showed the highest contents for bioactive compounds and higher potentially functional activities than other honeys. In contrast, viper bugloss samples showed the lowest bioactive compounds’ quantities and potentially functional activities regarding raw honeys, methanolic extracts and digests.

In general, honeys’ methanolic extracts showed lower total phenols and flavonoids, lower antioxidant and antimicrobial activities and higher anti-inflammatory activity than raw honeys. However, soluble fraction after in vitro digestion showed higher total phenolics and antioxidant activities and lower anti-inflammatory activity. These results suggest that some bioactive compounds of honey are resistant to the action of digestive enzymes or might be released during in vitro digestion, increasing the antioxidant and antimicrobial properties, whereas other compounds, such as flavonoids, could be degraded, so that other biological properties, such as anti-inflammatory activity, could decrease.

This study is the first where the oral phase in digestion of honey has been simulated. Observed differences between our study and previous research could highlight the necessity of including the oral phase in further in vitro digestion studies.

Our work confirms that, after in vitro gastrointestinal digestion, honey still keeps compounds with antioxidant and antimicrobial activity against *L. monocytogenes* and *S. aureus.*

Methanolic extracts obtained from honey can be useful for cosmetics and pharmaceuticals due to their anti-inflammatory activity. Digested honey still possesses important biological properties and can act as an antioxidant and antimicrobial in the human body.

## Figures and Tables

**Table 1:** Botanical origin of the honey samples.

Honey Samples	Botanical Origin	Cientific Name
H1	Honeydew	
H2	Honeydew	
H3	Honeydew	
H4	Honeydew	
H5	Viper’s bugloss/Blueweed	*Echium* sp.
H6	Viper’s bugloss/Blueweed	*Echium* sp.
H7	Viper’s bugloss/Blueweed	*Echium* sp.
H8	Ling heather	*Calluna vulgaris* (L.) Hull
H9	Ling heather	*Calluna vulgaris* (L.) Hull
H10	Ling heather	*Calluna vulgaris* (L.) Hull
H11	Ling heather	*Calluna vulgaris* (L.) Hull
H12	Ling heather	*Calluna vulgaris* (L.) Hull
H13	Broom	Leguminosae *Genista* sp.
H14	Broom	Leguminosae *Genista* sp.
H15	Broom	Leguminosae *Genista* sp.

**Table 2:** Minimal inhibition concentration (MIC) and minimal bactericidal concentration (MBC) of raw honeys (RH), methanolic honey extracts (ME) and honey after in vitro digestion (DH) expressed as % of honey, extract or digestive fraction and between brackets the corresponding honey concentration (g/100 mL). Triplicated showed identical MIC or MBC for each sample. Different MIC or MBC values indicate significant differences (*p* < 0.05) between samples with the same extraction for each microorganism.

***E. coli***																
		**H1**	**H2**	**H3**	**H4**	**H5**	**H6**	**H7**	**H8**	**H9**	**H10**	**H11**	**H12**	**H13**	**H14**	**H15**
RH	MIC	18.75 (18.75)	37.5 (37.5)	18.75 (18.75)	9.38 (9.38)	18.75 (18.75)	9.38 (9.38)	18.75 (18.75)	18.75 (18.75)	9.38 (9.38)	18.75 (18.75)	9.38 (9.38)	9.38 (9.38)	9.38 (9.38)	9.38 (9.38)	18.75 (18.75)
	MBC	18.75 (18.75)	>37.5 (>37.5)	18.75 (18.75)	9.38 (9.38)	18.75 (18.75)	9.38 (9.38)	18.75 (18.75)	18.75 (18.75)	18.75 (18.75)	37.5 (37.5)	18.75 (18.75)	9.38 (9.38)	9.38 (9.38)	9.38 (9.38)	18.75 (18.75)
ME	MIC	5 (25)	5 (25)	5 (25)	5 (25)	5 (25)	5 (25)	5 (25)	15 (75)	5 (25)	5 (25)	5 (25)	5 (25)	5 (25)	5 (25)	5 (25)
	MBC	5 (25)	5 (25)	5 (25)	5 (25)	5 (25)	5 (25)	5 (25)	15 (75)	5 (25)	5 (25)	5 (25)	5 (25)	5 (25)	5 (25)	5 (25)
DH	MIC	>90 (>7)	>90 (>7)	>90 (>7)	>90 (>7)	>90 (>7)	>90 (>7)	>90 (>7)	>90 (>7)	>90 (>7)	>90 (>7)	>90 (>7)	>90 (>7)	>90 (>7)	>90 (>7)	>90 (>7)
	MBC	>90 (>7)	>90 (>7)	>90 (>7)	>90 (>7)	>90 (>7)	>90 (>7)	>90 (>7)	>90 (>7)	>90 (>7)	>90 (>7)	>90 (>7)	>90 (>7)	>90 (>7)	>90 (>7)	>90 (>7)
***S. aureus***																
		**H1**	**H2**	**H3**	**H4**	**H5**	**H6**	**H7**	**H8**	**H9**	**H10**	**H11**	**H12**	**H13**	**H14**	**H15**
RH	MIC	9.38 (9.38)	>37.5 (>37.5)	9.38 (9.38)	4.69 (4.69)	9.38 (9.38)	4.69 (4.69)	9.38 (9.38)	9.38 (9.38)	9.38 (9.38)	9.38 (9.38)	4.69 (4.69)	9.38 (9.38)	4.69 (4.69)	4.69 (4.69)	9.38 (9.38)
	MBC	9.38 (9.38)	>37.5 (>37.5)	9.38 (9.38)	4.69 (4.69)	9.38 (9.38)	4.69 (4.69)	9.38 (9.38)	9.38 (9.38)	9.38 (9.38)	9.38 (9.38)	4.69 (4.69)	9.38 (9.38)	4.69 (4.69)	4.69 (4.69)	9.38 (9.38)
ME	MIC	15 (75)	5 (25)	5 (25)	5 (25)	5 (25)	15 (75)	5 (25)	5 (25)	5 (25)	5 (25)	5 (25)	5 (25)	5 (25)	5 (25)	5 (25)
	MBC	15 (75)	15 (75)	15 (75)	15 (75)	15 (75)	15 (75)	15 (75)	15 (75)	15 (75)	15 (75)	15 (75)	15 (75)	15 (75)	15 (75)	15 (75)
DH	MIC	40 (3.13)	<5 (<0.39)	<5 (<0.39)	<5 (<0.39)	<5 (<0.39)	12.5 (0.98)	25 (1.95)	25 (1.95)	25 (1.95)	<5 (<0.39)	25 (1.95)	25 (1.95)	25 (1.95)	<5 (<0.39)	25 (1.95)
	MBC	>90 (>7)	25 (1.95)	25 (1.95)	25 (1.95)	25 (1.95)	25 (1.95)	40 (3.13)	>90 (7)	25 (1.95)	25 (1.95)	25 (1.95)	25 (1.95)	75 (5.85)	25 (1.95)	90 (7)
***L. monocytogenes***																
		**H1**	**H2**	**H3**	**H4**	**H5**	**H6**	**H7**	**H8**	**H9**	**H10**	**H11**	**H12**	**H13**	**H14**	**H15**
RH	MIC	18.75 (18.75)	37.5 (37.5)	18.75 (18.75)	18.75 (18.75)	18.75 (18.75)	18.75 (18.75)	18.75 (18.75)	18.75 (18.75)	18.75 (18.75)	18.75 (18.75)	18.75 (18.75)	18.75 (18.75)	18.75 (18.75)	18.75 (18.75)	18.75 (18.75)
	MBC	37.5 (37.5)	>37.5 (37.5)	37.5 (37.5)	18.75 (18.75)	37.5 (37.5)	37.5 (37.5)	37.5 (37.5)	18.75 (18.75)	18.75 (18.75)	37.5 (37.5)	18.75 (18.75)	37.5 (37.5)	18.75 (18.75)	18.75 (18.75)	18.75 (18.75)
ME	MIC	5 (25)	<1 (<5)	<1 (<5)	5 (25)	<1 (<5)	5 (25)	5 (25)	<1 (<5)	5 (25)	<1 (<5)	5 (25)	5 (25)	<1 (<5)	<1 (<5)	5 (25)
	MBC	15 (75)	<1 (<1)	<1 (<1)	15 (75)	<1 (<1)	15 (75)	15 (75)	15 (75)	15 (75)	5 (25)	15 (75)	15 (75)	5 (25)	<1 (<1)	15 (75)
DH	MIC	40 (3.13)	40 (3.13)	40 (3.13)	40 (3.13)	40 (3.13)	40 (3.13)	40 (3.13)	40 (3.13)	40 (3.13)	40 (3.13)	>90 (>7)	>90 (>7)	40 (3.13)	40 (3.13)	40 (3.13)
	MBC	>90 (>7)	>90 (>7)	>90 (>7)	>90 (>7)	>90 (>7)	>90 (>7)	>90 (>7)	>90 (>7)	40 (3.13)	>90 (>7)	>90 (>7)	>90 (>7)	>90 (>7)	>90 (>7)	>90 (>7)
***St. mutans***																
		**H1**	**H2**	**H3**	**H4**	**H5**	**H6**	**H7**	**H8**	**H9**	**H10**	**H11**	**H12**	**H13**	**H14**	**H15**
RH	MIC	18.75 (18.75)	37.5 (37.5)	18.75 (18.75)	18.75 (18.75)	18.75 (18.75)	18.75 (18.75)	18.75 (18.75)	18.75 (18.75)	18.75 (18.75)	18.75 (18.75)	18.75 (18.75)	18.75 (18.75)	18.75 (18.75)	18.75 (18.75)	18.75 (18.75)
	MBC	18.75 (18.75)	37.5 (37.5)	18.75 (18.75)	18.75 (18.75)	37.5 (37.5)	18.75 (18.75)	18.75 (18.75)	18.75 (18.75)	18.75 (18.75)	37.5 (37.5)	18.75 (18.75)	18.75 (18.75)	18.75 (18.75)	18.75 (18.75)	18.75 (18.75)
ME	MIC	15 (75)	15 (75)	15 (75)	15 (75)	15 (75)	15 (75)	15 (75)	15 (75)	15 (75)	15 (75)	15 (75)	5 (25)	15 (75)	15 (75)	15 (75)
	MBC	15 (75)	15 (75)	15 (75)	15 (75)	15 (75)	15 (75)	15 (75)	15 (75)	15 (75)	15 (75)	15 (75)	15 (75)	15 (75)	15 (75)	15 (75)
DH	MIC	>90 (>7)	>90 (>7)	>90 (>7)	>90 (>7)	>90 (>7)	>90 (>7)	>90 (>7)	>90 (>7)	>90 (>7)	>90 (>7)	>90 (>7)	>90 (>7)	>90 (>7)	>90 (>7)	>90 (>7)
	MBC	>90 (>7)	>90 (>7)	>90 (>7)	>90 (>7)	>90 (>7)	>90 (>7)	>90 (>7)	>90 (>7)	>90 (>7)	>90 (>7)	>90 (>7)	>90 (>7)	>90 (>7)	>90 (>7)	>90 (>7)
***C. albicans***																
		**H1**	**H2**	**H3**	**H4**	**H5**	**H6**	**H7**	**H8**	**H9**	**H10**	**H11**	**H12**	**H13**	**H14**	**H15**
RH	MIC	37.5 (37.5)	37.5 (37.5)	37.5 (37.5)	37.5 (37.5)	37.5 (37.5)	37.5 (37.5)	37.5 (37.5)	37.5 (37.5)	37.5 (37.5)	37.5 (37.5)	37.5 (37.5)	37.5 (37.5)	37.5 (37.5)	37.5 (37.5)	37.5 (37.5)
	MBC	>45 (>45)	>45 (>45)	>45 (>45)	>45 (>45)	>45 (>45)	>45 (>45)	>45 (>45)	>45 (>45)	>45 (>45)	>45 (>45)	>45 (>45)	>45 (>45)	>45 (>45)	45 (>45)	>45 (>45)
ME	MIC	15 (75)	15 (75)	15 (75)	15 (75)	15 (75)	15 (75)	15 (75)	15 (75)	15 (75)	15 (75)	15 (75)	15 (75)	15 (75)	15 (75)	15 (75)
	MBC	15 (75)	15 (75)	15 (75)	15 (75)	15 (75)	15 (75)	15 (75)	15 (75)	15 (75)	15 (75)	15 (75)	15 (75)	15 (75)	15 (75)	15 (75)
DH	MIC	>90 (>7)	>90 (>7)	>90 (>7)	>90 (>7)	>90 (>7)	>90 (>7)	>90 (>7)	>90 (>7)	>90 (>7)	>90 (>7)	>90 (>7)	>90 (>7)	>90 (>7)	>90 (>7)	>90 (>7)
	MBC	>90 (>7)	>90 (>7)	>90 (>7)	>90 (>7)	>90 (>7)	>90 (>7)	>90 (>7)	>90 (>7)	>90 (>7)	>90 (>7)	>90 (>7)	>90 (>7)	>90 (>7)	>90 (>7)	>90 (>7)
***Ps. aeruginosa***																
		**H1**	**H2**	**H3**	**H4**	**H5**	**H6**	**H7**	**H8**	**H9**	**H10**	**H11**	**H12**	**H13**	**H14**	**H15**
RH	MIC	9.38 (9.38)	37.5 (37.5)	9.38 (9.38)	9.38 (9.38)	9.38 (9.38)	9.38 (9.38)	9.38 (9.38)	9.38 (9.38)	9.38 (9.38)	9.38 (9.38)	9.38 (9.38)	9.38 (9.38)	9.38 (9.38)	9.38 (9.38)	9.38 (9.38)
	MBC	9.38 (9.38)	37.5 (37.5)	9.38 (9.38)	9.38 (9.38)	9.38 (9.38)	9.38 (9.38)	9.38 (9.38)	9.38 (9.38)	9.38 (9.38)	9.38 (9.38)	9.38 (9.38)	9.38 (9.38)	9.38 (9.38)	9.38 (9.38)	9.38 (9.38)
ME	MIC	5 (25)	5 (25)	5 (25)	5 (25)	5 (25)	5 (25)	5 (25)	5 (25)	5 (25)	5 (25)	5 (25)	5 (25)	5 (25)	5 (25)	5 (25)
	MBC	5 (25)	5 (25)	5 (25)	5 (25)	5 (25)	5 (25)	5 (25)	5 (25)	5 (25)	5 (25)	5 (25)	5 (25)	5 (25)	5 (25)	5 (25)
DH	MIC	>90 (>7)	>90 (>7)	>90 (>7)	>90 (>7)	>90 (>7)	>90 (>7)	>90 (>7)	>90 (>7)	>90 (>7)	>90 (>7)	>90 (>7)	>90 (>7)	>90 (>7)	>90 (>7)	>90 (>7)
	MBC	>90 (>7)	>90 (>7)	>90 (>7)	>90 (>7)	>90 (>7)	>90 (>7)	>90 (>7)	>90 (>7)	>90 (>7)	>90 (>7)	>90 (>7)	>90 (>7)	>90 (>7)	>90 (>7)	>90 (>7)

## Data Availability

The data presented in this study are available on request from the corresponding authors.
